# Supplementary material for: Promoting high-voltage stability through local lattice distortion of halide solid electrolytes
Source: Nat Commun. 2024 Feb 17;15:1481. doi: 10.1038/s41467-024-45864-1 (PMC10874449; doi:10.1038/s41467-024-45864-1)
Supplement: Supplementary file 1 — Supplementary Information [file 41467_2024_45864_MOESM1_ESM.pdf]

Promoting high-voltage stability through local lattice distortion of halide solid electrolytes

Zhenyou Song[1,9], Tengrui Wang[1,9], Hua Yang[2,3,9], Wang Hay Kan[2,3]*, Yuwei Chen[1], Qian Yu[1], Likuo Wang[1], Yini Zhang[1], Yiming Dai[1], Huaican Chen[2,3], Wen Yin[2,3], Takashi Honda[4,5], Maxim Avdeev[6,7], Henghui Xu[8], Jiwei Ma[1], Yunhui Huang[8]* and Wei Luo[1]*

[1]Institute of New Energy for Vehicles, School of Materials Science and Engineering, Tongji University, Shanghai 201804, China;

[2]Spallation Neutron Source Science Center, Dongguan, Guangdong 523803, China;

[3]Institute of High Energy Physics, Chinese Academy of Sciences, Beijing 100049, China;

[4]Materials & Life Science Division, J-PARC Centre, Tokai, Ibaraki, Japan;

[5]Institute of Materials Structure Science, High Energy Accelerator Research Organization (KEK), Tsukuba, Ibaraki, Japan;

[6]Australian Nuclear Science and Technology Organization (ANSTO), Lucas Heights, NSW, 2234, Australia;

[7]Department of Chemistry, University of Sydney, Sydney, NSW, 2006, Australia;

[8]State Key Laboratory of Material Processing and Die & Mould Technology, School of Materials Science and Engineering, Huazhong University of Science and Technology, Wuhan, Hubei 430074, China;

[9]These authors contributed equally: Zhenyou Song, Tengrui Wang, Hua Yang.

*Email: weiluo@tongji.edu.cn, jianhx@ihep.ac.cn, and huangyh@hust.edu.cn

Calculation of configurational entropy: Similar to the concepts of high-entropy alloys (HEAs) and high-entropy oxides (HEOs), a unified definition of high-entropy halide SEs can be described as single-phase halide systems containing 5 or more cations. The S_{config} for the $Li_xM_yCl_z$ type halide can be calculated by the following formula:

$$S_{\mathrm{config}} = -R\left[x\left(\sum_{a=1}^{M} x_a \ln x_a\right)_{\mathrm{Li-site}} + y\left(\sum_{b=1}^{N} y_b \ln y_b\right)_{\mathrm{M-site}} + z\left(\sum_{c=1}^{P} z_c \ln z_c\right)_{\mathrm{Cl-site}}\right] \quad (1)$$

where x_a, y_b, and z_c are the mole fractions of components in cation sites and anion sites and M, N, and P are ion numbers, respectively.

For Li_nMCl_{3+n} type halide SEs, the formula can be modified as follows.

For HE-LIC ($Li_{2.75}Y_{0.16}Er_{0.16}Yb_{0.16}In_{0.25}Zr_{0.25}Cl_6$),

$$S_{\mathrm{config}} = -R\left[\left(\sum_{b=1}^{N} y_b \ln y_b\right)_{\mathrm{M-site}}\right] = -R\left(3\times\frac{1}{6}\ln\frac{1}{6} + 2\times\frac{1}{4}\ln\frac{1}{4}\right) = 1.589R \quad (2)$$

For ME-LIC ($Li_{2.75}Y_{0.5}In_{0.25}Zr_{0.25}Cl_6$),

$$S_{\mathrm{config}} = -R\left[\left(\sum_{b=1}^{N} y_b \ln y_b\right)_{\mathrm{M-site}}\right] = -R\left(\frac{1}{2}\ln\frac{1}{2} + 2\times\frac{1}{4}\ln\frac{1}{4}\right) = 1.040R \quad (3)$$

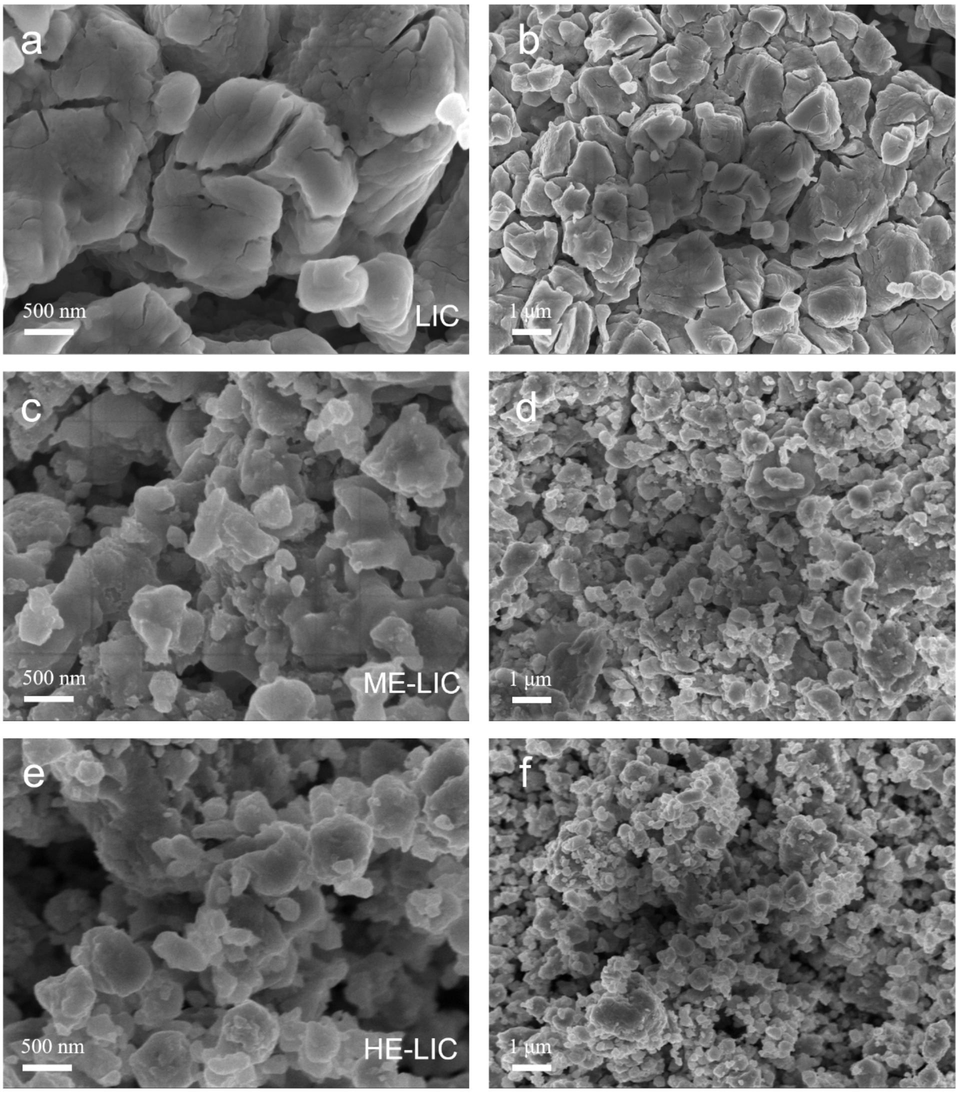

Supplementary Figure 1. SEM images of samples: **a, b** LIC, **c, d** ME-LIC and **e, f** HE-LIC.

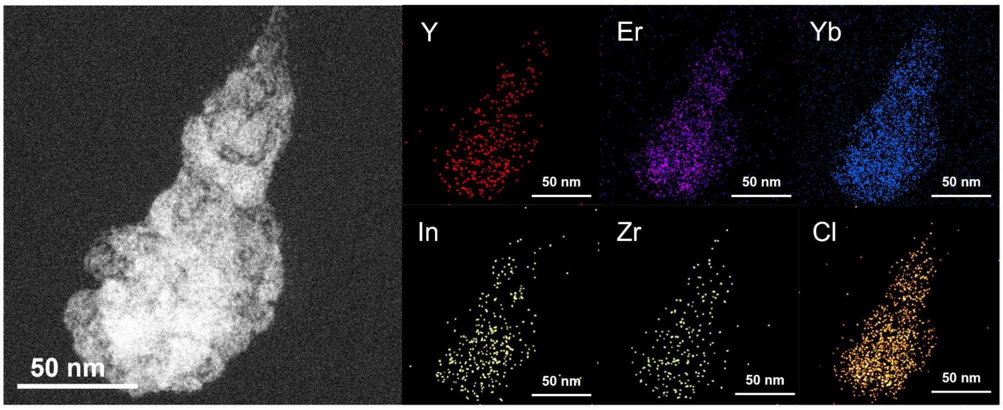

Supplementary Figure 2. STEM-EDX for HE-LIC, showing uniform elemental distribution on the nanometer level.

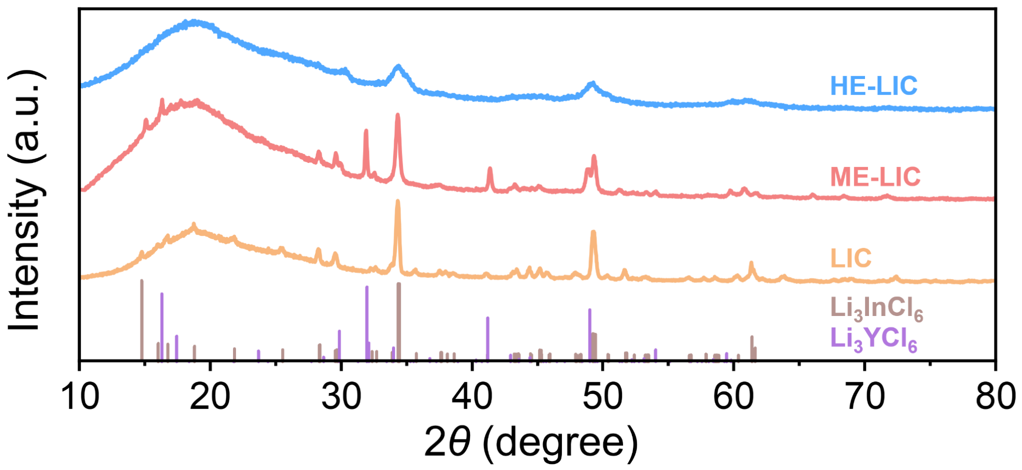

Supplementary Figure 3. X-ray diffraction patterns of LIC, ME-LIC and HE-LIC.

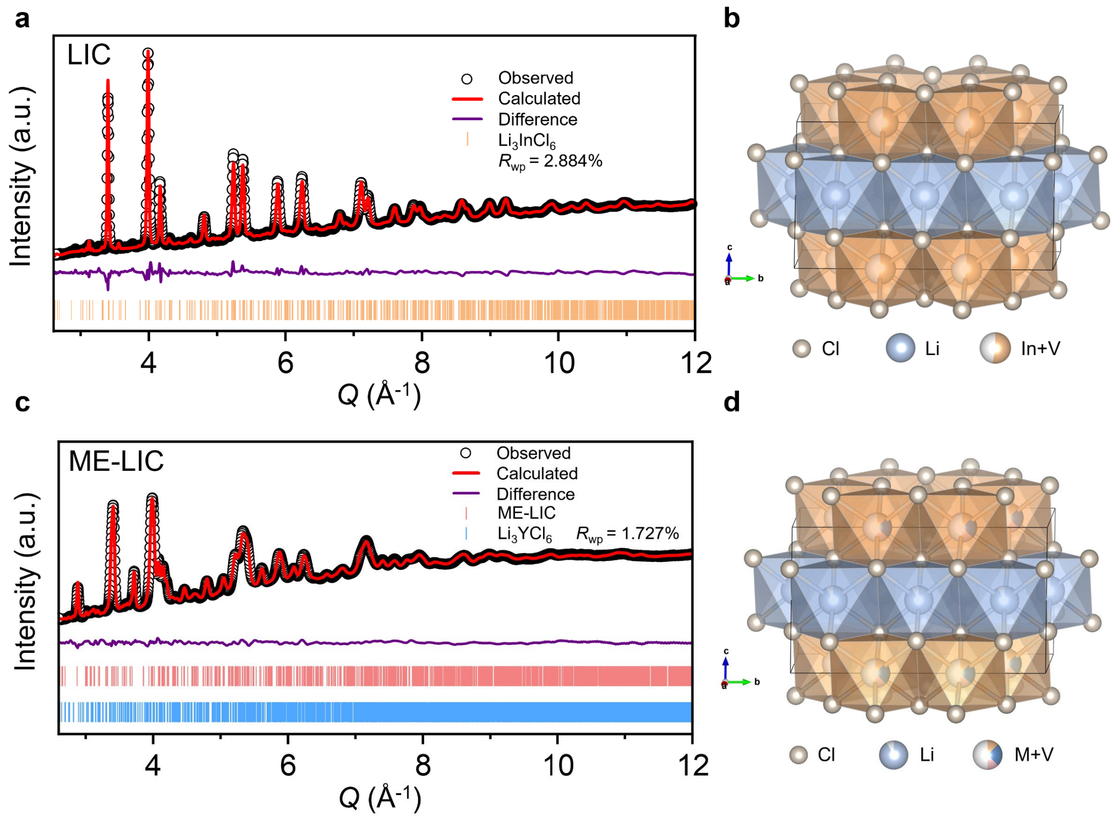

Supplementary Figure 4. Neutron diffraction patterns and the corresponding refinements of **a, b** LIC and **c, d** ME-LIC, with corresponding graphical representations of unit cells from the refinements.

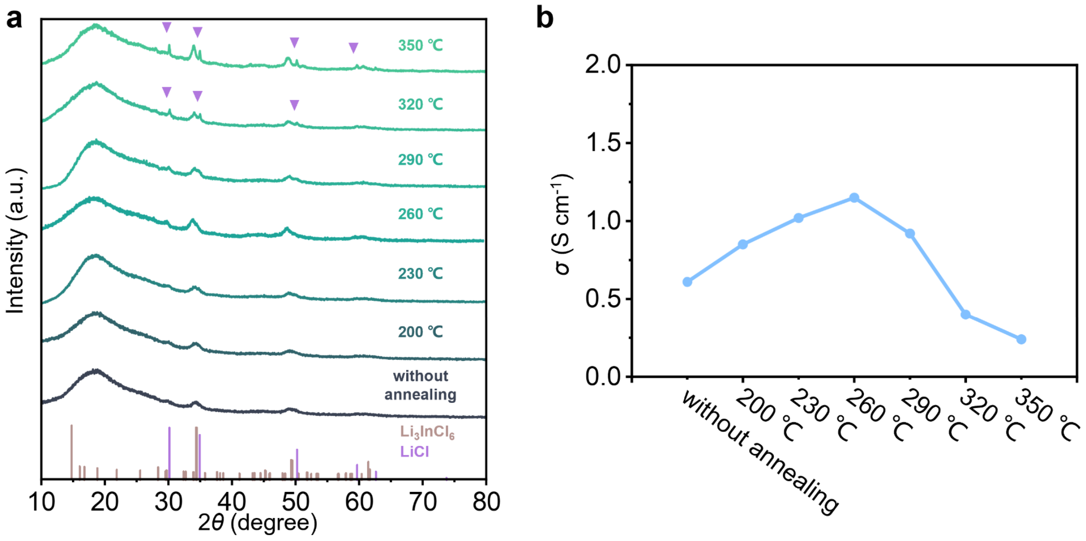

Supplementary Figure 5. a XRD patterns and **b** Li^+ conductivities of HE-LIC annealed at different temperatures.

Supplementary Discussion 1. XRD patterns and Li^+ ions conductivities of HE-LIC annealed at different temperatures are shown in **Supplementary Fig. 5**. When temperature is below 260 °C, the crystallinity and Li^+ conductivity of HE-LIC increase as the heating temperature rises. But above 260 °C, distinct impurity phase of LiCl is formed, suggesting the decomposition of HE-LIC and leading to a decrease of Li^+ conductivity. Furthermore, confirmed by ND results in **Supplementary Fig. 6**, the sample without annealing exhibits a pure phase without LiCl, revealing that LiCl is produced at high temperature.

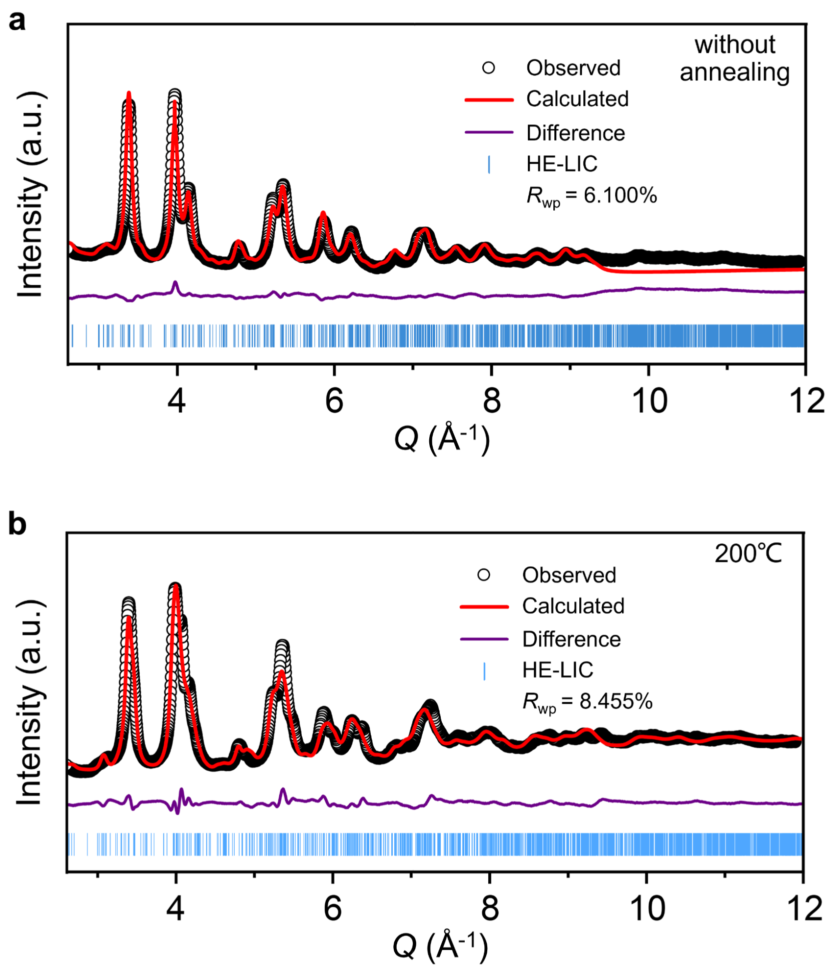

Supplementary Figure 6. Neutron diffraction patterns and the corresponding refinements of HE-LIC **a** without annealing and **b** annealed at 200 °C.

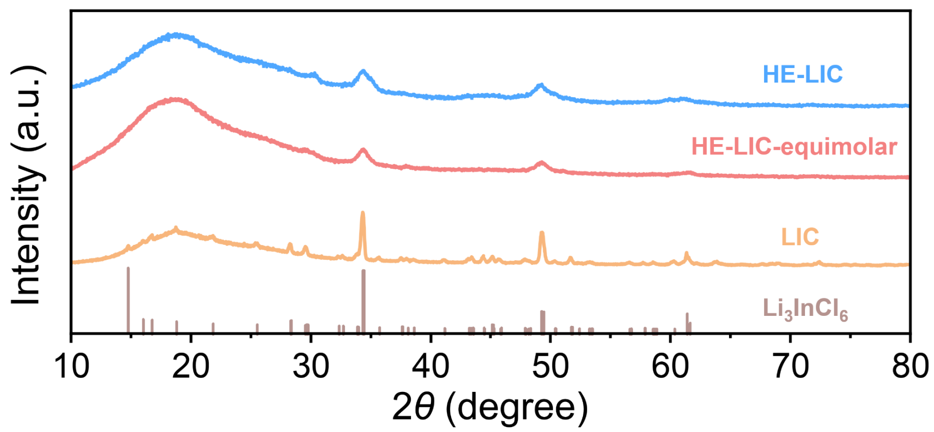

Supplementary Figure 7. X-ray diffraction patterns of HE-LIC-equimolar, HE-LIC and LIC.

Supplementary Discussion 2. At first, we have synthesized equimolar $Li_{2.8}Y_{0.2}Er_{0.2}Yb_{0.2}In_{0.2}Zr_{0.2}Cl_6$ (HE-LIC-equimolar), which exhibits similar XRD patterns as the non-equimolar $Li_{2.75}Y_{0.16}Er_{0.16}Yb_{0.16}In_{0.25}Zr_{0.25}Cl_6$ (HE-LIC), as depicted in **Supplementary Figure 7**.

The HE-LIC-equimolar exhibits a Li^+ conductivity of 0.930 mS cm^{-1} and an activation energy of 0.352 eV (**Supplementary Figure 8.**). Furthermore, we have discovered that by slightly adjusting the proportion of certain elements on this basis, we can further enhance the electrochemical performance. For instance, incorporating more quadrivalent Zr results in additional lithium vacancies. With more In, it can exhibit enhanced ionic conductivity in heat treatment compared to Y, Er, and Yb. Meanwhile, we have to ensure that the configurational entropy is not significantly compromised. Therefore, we reach the non-equimolar formula $Li_{2.75}Y_{0.16}Er_{0.16}Yb_{0.16}In_{0.25}Zr_{0.25}Cl_6$. As expected, this composition demonstrates an ionic conductivity of 1.171 mS cm^{-1} and a lower activation energy (0.338 eV) compared to HE-LIC-equimolar. Therefore, we selected HE-LIC as the primary focus of this study.

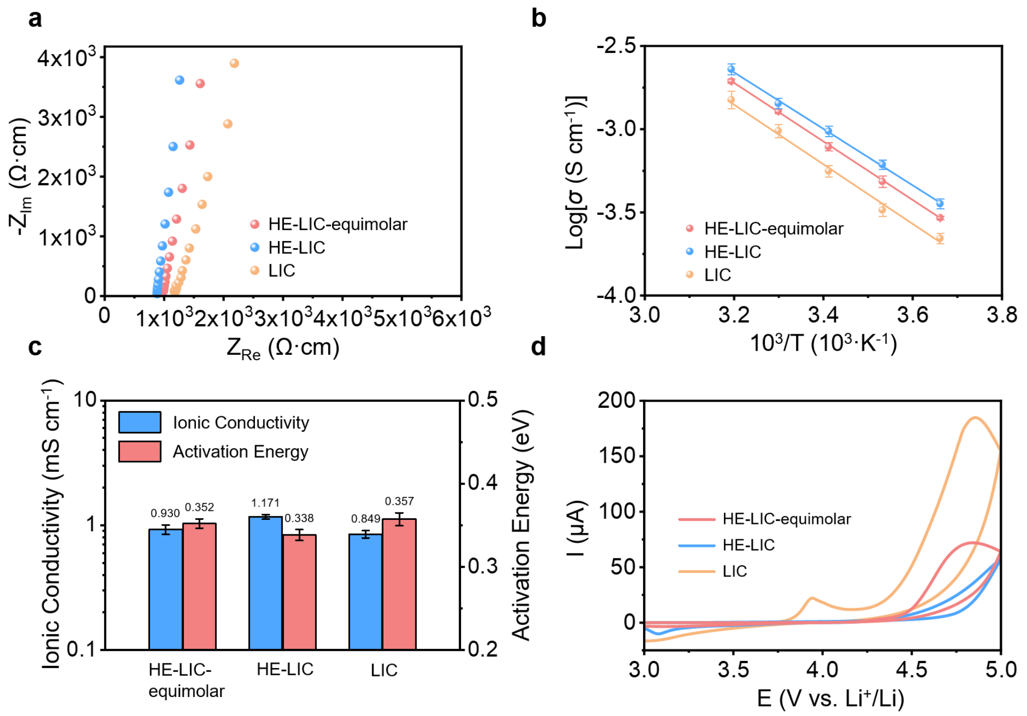

Supplementary Figure 8. Electrochemical properties comparison of HE-LIC-equimolar, HE-LIC and LIC. **a** Typical Nyquist plots at room temperature, normalized for the pellet thickness and area. **b** Arrhenius conductivity plots. **c** Summary of electrochemical properties. **d** CV curves within 3.0 and 5.0 V vs. Li^+/Li.

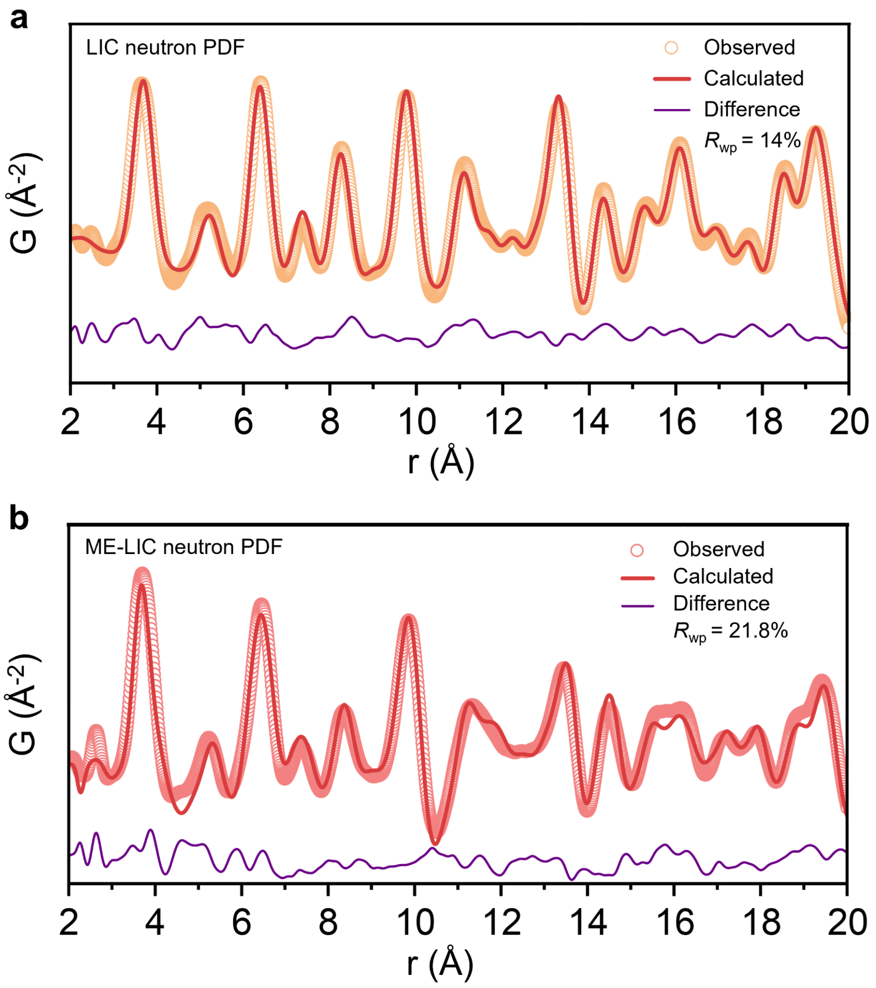

Supplementary Figure 9. PDF refinement of **a** LIC and **b** ME-LIC for the atomic paired radial distribution with the lowest energy supercell as the structural model. A two-phase refinement approach was adopted to ME-LIC, incorporating *Pnma*-type Li_3YCl_6.

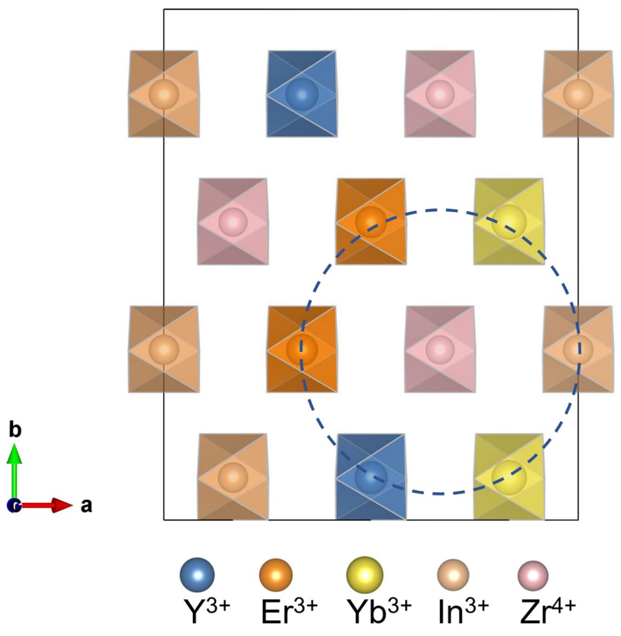

Supplementary Figure 10. The occupancy of Y^{3+}, Er^{3+}, Yb^{3+}, In^{3+} and Zr^{4+} at the *4g* site in the lowest energy supercell with the selective location of Zr^{4+} in the structure.

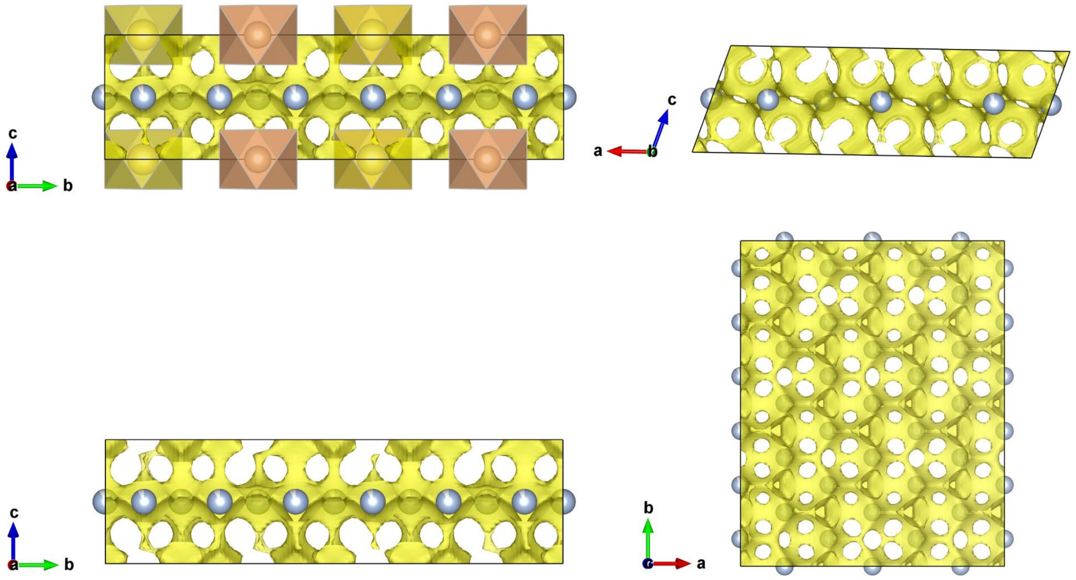

Supplementary Figure 11. The Li^+ probability density isosurface of the supercell based on BVEL, indicating the Li-ion migration paths in HE-LIC.

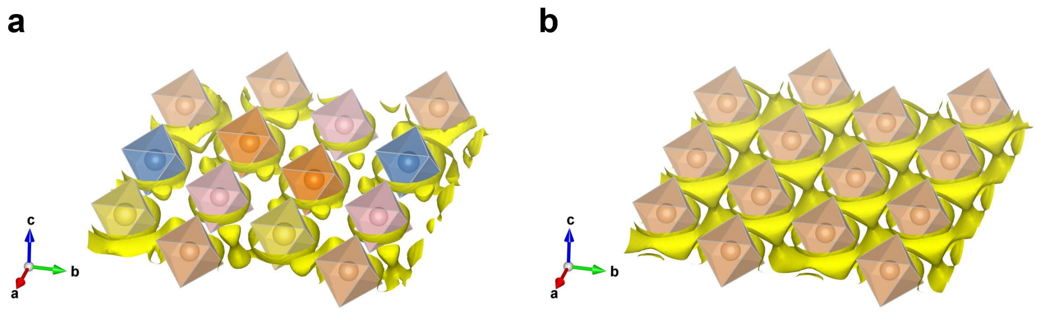

Supplementary Figure 12. The probability density isosurface of chloride ions of the supercell based on BVEL: **a** HE-LIC, **b** LIC.

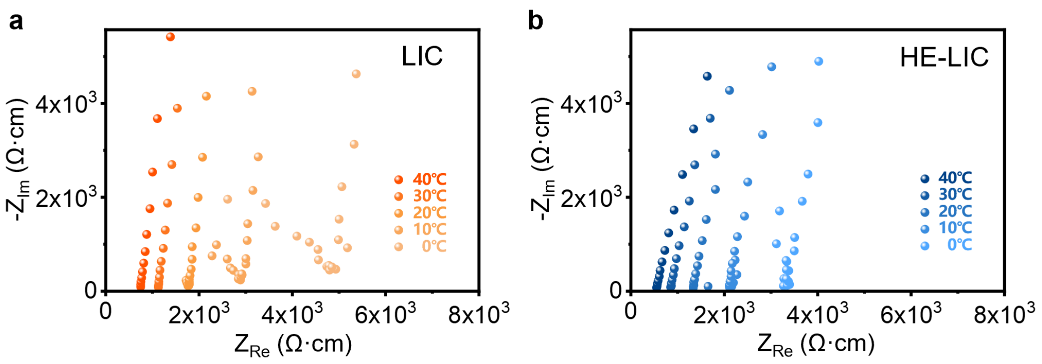

Supplementary Figure 13. Nyquist plots of **a** LIC and **b** HE-LIC at different temperatures from 0 to 40 °C.

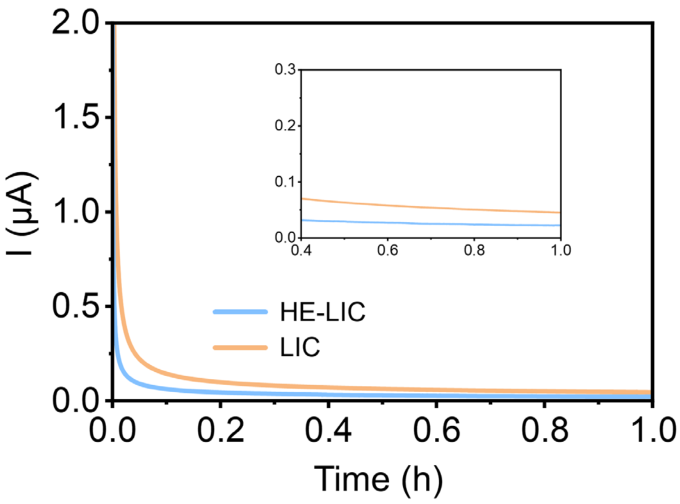

Supplementary Figure 14. Typical DC polarization curves with an applied voltage of 1 V, with the inset showing the stable polarization current.

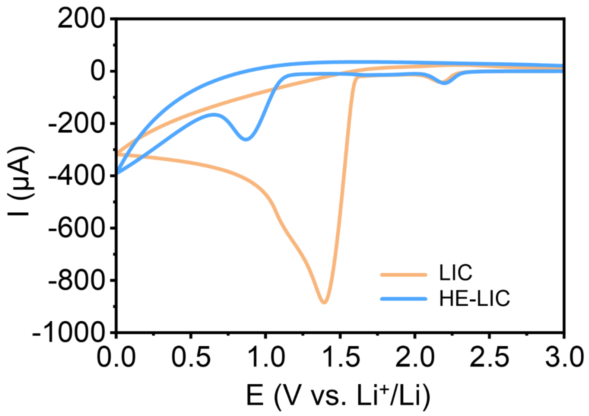

Supplementary Figure 15. CV curves of Li-In | LPSCl | halide SE | halide SE-VGCF cells within 0 and 3.0 V vs. Li^+/Li at a scanning rate of 0.1 mV s^{-1}.

Supplementary Discussion 3. To investigate the stability of halide solid electrolytes towards Li, we first performed CV tests on both LIC and HE-LIC in the voltage range of 0-3.0 V vs. Li^+/Li. **Supplementary Figure 15** illustrates that both LIC and HE-LIC exhibit a weak cathodic peak at approximately 2.2 V. This peak corresponds to the initiation of a weak reduction process, specifically the conversion from In^{3+} to In^{2+}. Notably, when the voltage drops below 1.6 V, LIC undergoes a significant reduction reaction, whereas HE-LIC experiences reduction below 1.1 V. Furthermore, the reduction reaction current of HE-LIC is considerably lower than that of LIC, indicating that HE-LIC demonstrates improved reduction stability.

To further assess the reduction stability of LIC and HE-LIC, we assembled symmetric cells with Li-In anodes (**Supplementary Figure 16**). The polarization voltage of the Li-In|LIC|Li-In symmetric cell exceeds 5 V, and the impedance exceeds 10000 Ω after just one cycle. HE-LIC exhibits improved cycling stability, as evidenced by a stable cycle lasting for 400 h. However, the polarization voltage remained above 0.8 V, and the impedance reached 4000 Ω after cycling, suggesting that HE-LIC is also unstable

to Li-In. So, we have chosen Li_6PS_5Cl (LPSCl) to prevent the reduction of halide electrolytes. LPSCl has exhibited excellent stability in Li-In symmetric cells, with a consistent and stable cycle lasting for 400 h. Notably, LPSCl demonstrated a polarization voltage below 0.01 V and an impedance of only 10 Ω.

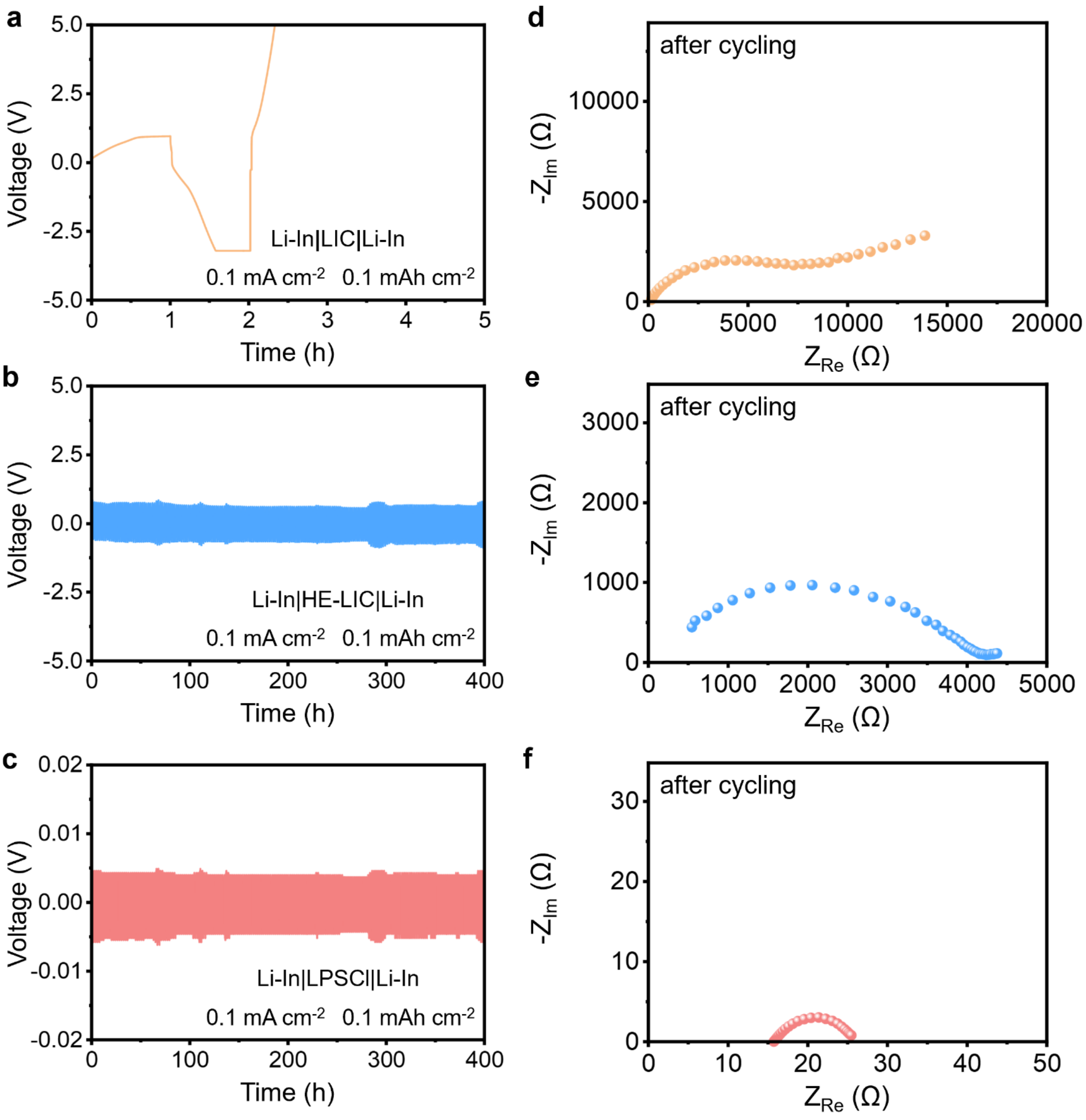

Supplementary Figure 16. Cycling performance of Li-In symmetric cells based on **a** LIC, **b** HE-LIC and **c** LPSCl, and **d, e, f** corresponding post-cycle impedance spectra.

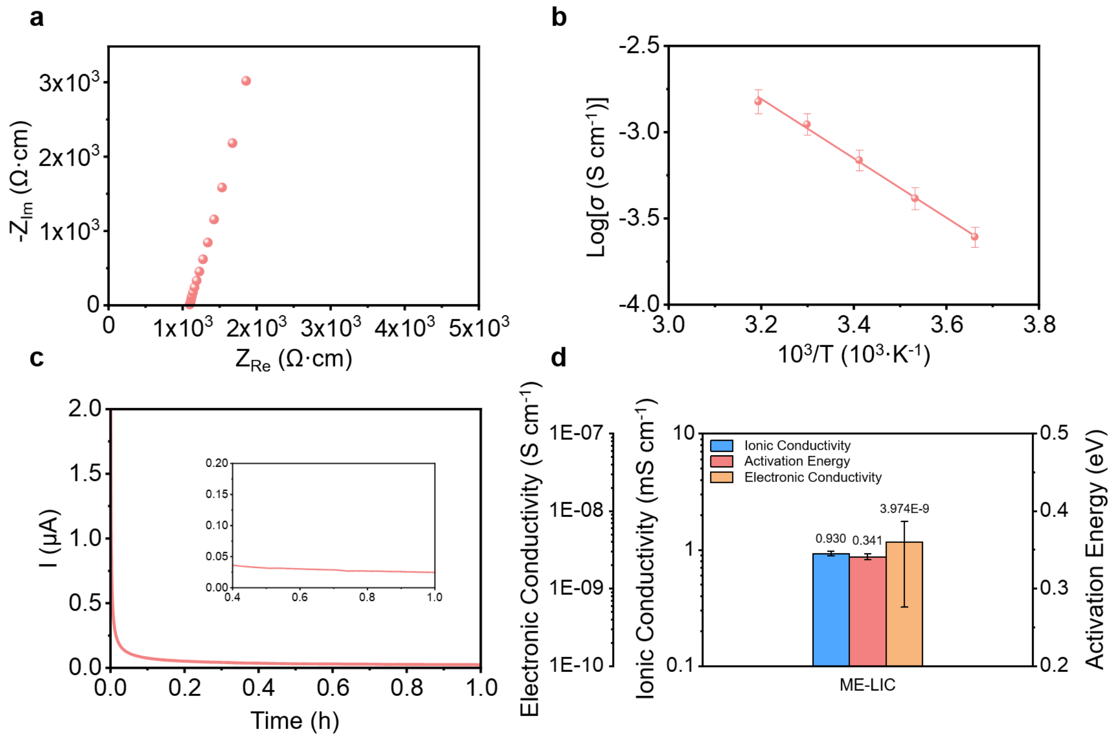

Supplementary Figure 17. Electrochemical properties of ME-LIC. **a** Typical Nyquist plots at room temperature, normalized for the pellet thickness and area. **b** Arrhenius conductivity plots. **c** DC polarization curves with an applied voltage of 1 V, with the inset showing the stable polarization current. **d** Summary of electrochemical properties.

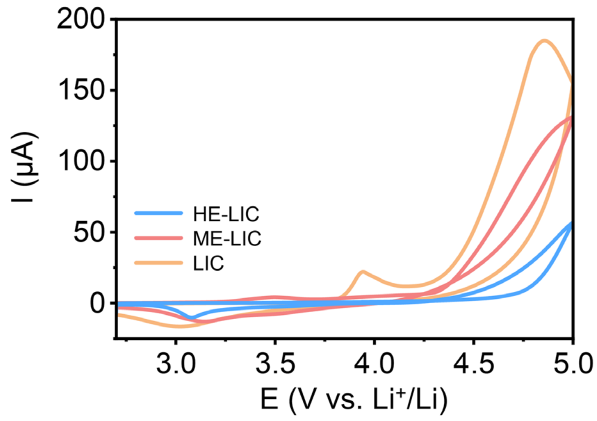

Supplementary Figure 18. CV curves of ME-LIC, compared with LIC and HE-LIC.

Supplementary Discussion 4. The XRD and neutron diffraction refining results of ME-LIC are shown in **Supplementary Figure 3, 4**. Obviously, ME-LIC is not a pure phase, which consists of a major phase (63 wt.%) isostructural with Li_3InCl_6 (*C2/m* structure) and a distinct second phase (37 wt.%) characterized as *Pnma*-type Li_3YCl_6. Furthermore, the neutron PDF analysis of the ME-LIC sample also confirmed this result (**Supplementary Figure 9**). As illustrated in **Supplementary Figure 17**, ME-LIC shows a Li^+ conductivity of 0.930 mS cm^{-1}, an activation energy of 0.341 eV and an electronic conductivity of 3.974×10^{-9} S cm^{-1}. Furthermore, the CV test exhibits a comparable oxidation potential of 4.28 V as LIC (**Supplementary Figure 18**). However, it should be noted that that the electrochemical properties of ME-LIC may not accurately reflect the performance of the target product, $Li_{2.75}Y_{0.5}In_{0.25}Zr_{0.25}Cl_6$, due to the significant presence of the second phase.

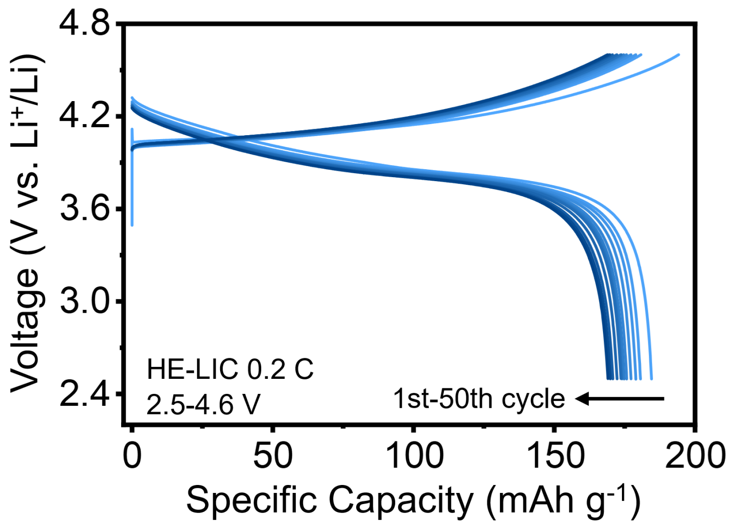

Supplementary Figure 19. Charge-discharge curves of the HE-LIC ASSB at 0.2 C within a voltage window of 2.5-4.6 V, corresponding to cycling performances in **Fig. 4h**.

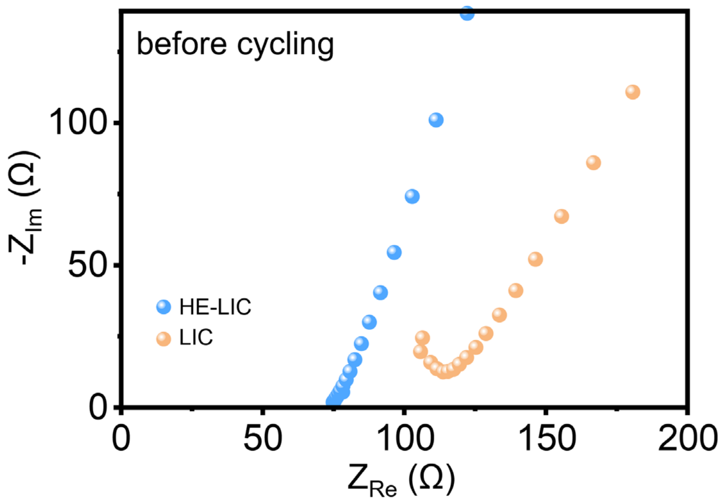

Supplementary Figure 20. Impedance measurements of ASSBs from 1 MHz to 0.1 Hz before cycling.

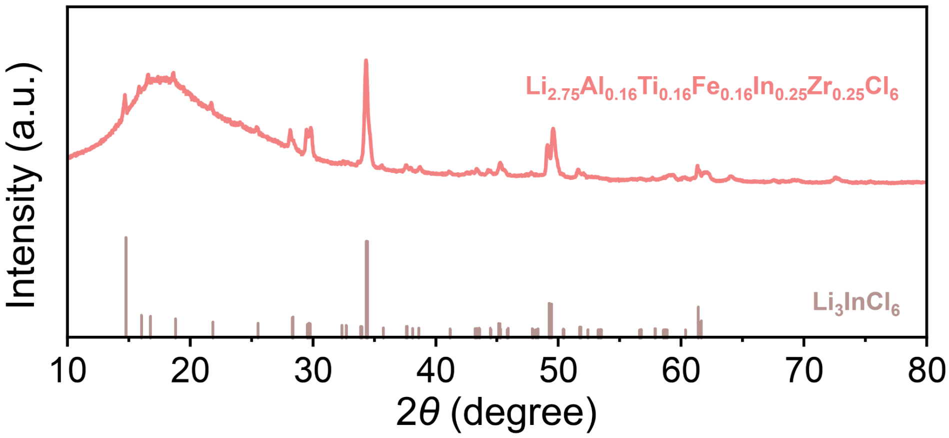

Supplementary Figure 21. XRD pattern of $Li_{2.75}Al_{0.16}Ti_{0.16}Fe_{0.16}In_{0.25}Zr_{0.25}Cl$.

Supplementary Discussion 5. We further measure the electrochemical properties of $Li_{2.75}Al_{0.16}Ti_{0.16}Fe_{0.16}In_{0.25}Zr_{0.25}Cl_6$, which exhibits a Li^+ conductivity of 0.454 mS cm^{-1}, an activation energy of 0.395 eV and an electronic conductivity of 3.633×10^{-9} S cm^{-1} (**Supplementary Figure 22**). We then also assembled Li-In | LPSCl | $Li_{2.75}Al_{0.16}Ti_{0.16}Fe_{0.16}In_{0.25}Zr_{0.25}Cl_6$ | LCO full cells. **Supplementary Figure 23** presents the initial cycle charge-discharge curves, where the $Li_{2.75}Al_{0.16}Ti_{0.16}Fe_{0.16}In_{0.25}Zr_{0.25}Cl_6$ cell delivers an initial discharge capacity of 109.4 mAh g^{-1} and an initial Coulombic efficiency (ICE) of 91.5%. These findings underscore the potential for further exploration in optimizing the performance of HE solid electrolytes based on abundant elements.

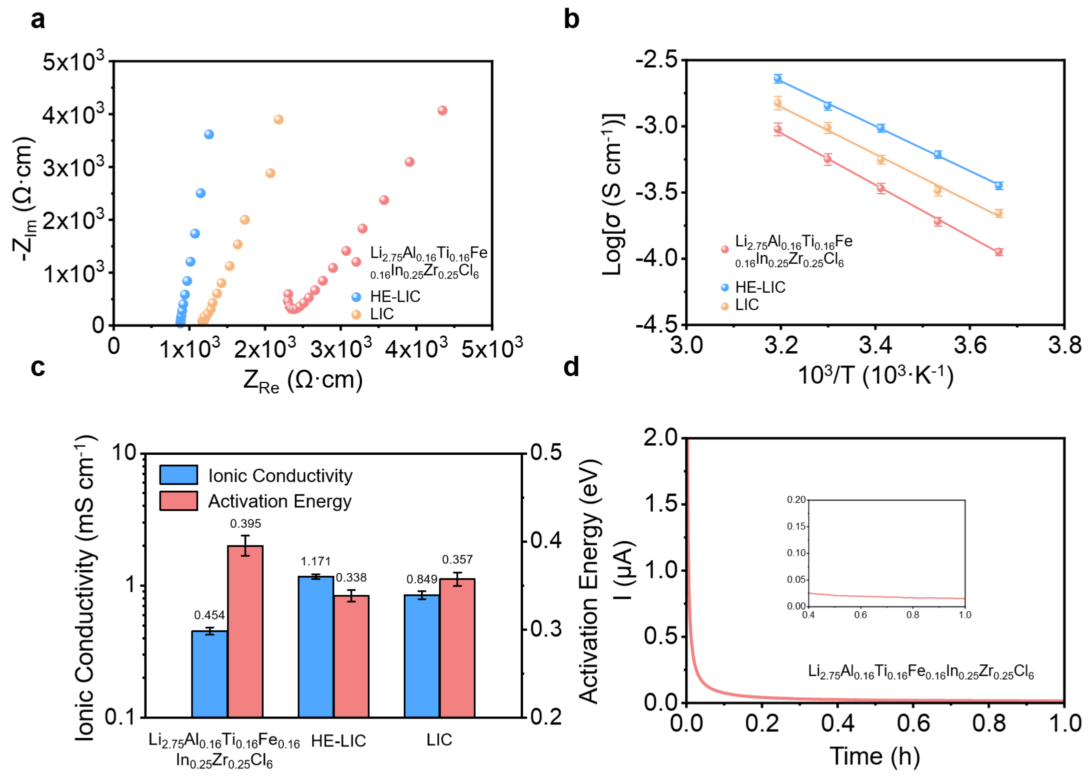

Supplementary Figure 22. Electrochemical properties of $Li_{2.75}Al_{0.16}Ti_{0.16}Fe_{0.16}In_{0.25}Zr_{0.25}Cl_6$, compared with HE-LIC and LIC. **a** Typical Nyquist plots at room temperature, normalized for the pellet thickness and area. **b** Arrhenius conductivity plots. **c** Summary of ionic conductivity and activation energy. **d** DC polarization curve of $Li_{2.75}Al_{0.16}Ti_{0.16}Fe_{0.16}In_{0.25}Zr_{0.25}Cl_6$ with an applied voltage of 1 V, with the inset showing the stable polarization current.

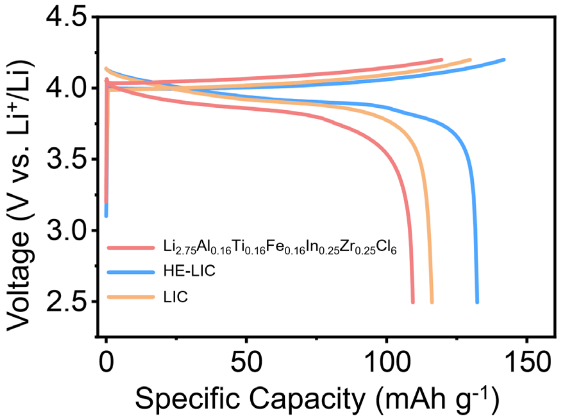

Supplementary Figure 23. The initial cycle charge–discharge curves of cells with three different halide SSEs tested at 0.5C.

Supplementary Table 1. Structural refinement details from the neutron diffraction data for HE-LIC.

$Li_{2.75}Y_{0.16}Er_{0.16}Yb_{0.16}In_{0.25}Zr_{0.25}Cl_6$ Space group: *C2/m*

a = 6.223(5) Å, b = 11.141(6) Å, c = 6.414(7) Å, β = 108.25(2)°

**Atom**	**x**	**y**	**z**	**Occupancy**	**Multiplicity**	**U_{iso} (Å^2)**
Cl1	0.266(2)	0	-0.254(2)	1	4	0.020(1)
Cl2	0.253(1)	0.172(1)	0.252(2)	1	8	0.020(1)
Y/Er/Yb/ In/Zr	0	0.33330	0	0.08/0.08/0.08/ 0.125/0.125	4	0.033(4)
Li1	0.5	0	0.5	0.9167	2	0.006(6)
Li2	0	0.204(3)	0.5	0.9167	4	0.006(6)

Supplementary Table 2. Structural refinement details from the neutron diffraction data for ME-LIC.

$Li_{2.75}Y_{0.5}In_{0.25}Zr_{0.25}Cl_6$ Space group: *C2/m*

a = 6.432(8) Å, b = 10.971(7) Å, c = 6.394(9) Å, β = 109.17(2)°

**Atom**	**x**	**y**	**z**	**Occupancy**	**Multiplicity**	**U_{iso} (Å^2)**
Cl1	0.253(2)	0	-0.254(2)	1	4	0.024(2)
Cl2	0.251(1)	0.159(2)	0.247(3)	1	8	0.024(2)
Y/In/Zr	0	0.33330	0	0.25/0.125/0.125	4	0.023(3)
Li1	0.5	0	0.5	0.9167	2	0.110(7)
Li2	0	0.164(2)	0.5	0.9167	4	0.110(7)

Supplementary Table 3. Structural refinement details from the neutron diffraction data for LIC.

Li_3InCl_6 Space group: *C2/m*						
a = 6.401(6) Å, b = 11.035(2) Å, c = 6.397(6) Å, β = 109.54(1)°						
**Atom**	**x**	**y**	**z**	**Occupancy**	**Multiplicity**	**U_{iso} (Å^2)**
Cl1	0.250(1)	0	-0.230(1)	1	4	0.0115(5)
Cl2	0.236(1)	0.1604(4)	0.243(7)	1	8	0.0115(5)
In1	0	0.33330	0	0.53	4	0.056(6)
Li1	0.5	0	0.5	1	2	0.104(9)
Li2	0	0.155(1)	0.5	1	4	0.104(9)

Supplementary Table 4. The positions of ions at 4g site in the lowest energy supercell.

Atom	x	y	z	Occupancy
In	0.00000	0.83335	0.00000	1
In	0.16667	0.08335	0.00000	1
In	0.00000	0.33335	0.00000	1
Y	0.33333	0.83335	0.00000	1
Y	0.50000	0.08335	0.00000	1
Er	0.33333	0.33335	0.00000	1
Er	0.50000	0.58335	0.00000	1
Yb	0.83333	0.08335	0.00000	1
Yb	0.83333	0.58335	0.00000	1
Zr	0.16667	0.58335	0.00000	1
Zr	0.66667	0.83335	0.00000	1
Zr	0.66667	0.33335	0.00000	1

Supplementary Table 5. The EIS spectra fitting results of Li-In||LPSCl/HE-LIC||LCO after cycling in **Fig. 5a**.

**Parameters**	**Value**	**Unit**	**Deviation**
$R_{SE,bulk+HF}$	55	Ohm	6
Q_{MF}	9.49e-5	$F{\cdot}s^{a-1}$	2.13e-5
a_{MF}	0.71	/	0.12
R_{MF}	47	Ohm	11
Q_{LF}	2.23e-3	$F{\cdot}s^{a-1}$	0.48e-3
a_{LF}	0.39	/	0.08
R_{LF}	17	Ohm	8
Q_{w}	3.65e-2	$F{\cdot}s^{a-1}$	1.62e-2
a_{w}	0.33	/	0.16

Supplementary Table 6. The EIS spectra fitting results of Li-In||LPSCl/LIC||LCO after cycling in **Fig. 5b**.

**Parameters**	**Value**	**Unit**	**Deviation**
$R_{SE,bulk}$	11	Ohm	64
Q_{HF}	4.02e-9	$F{\cdot}s^{a-1}$	4.86e-9
a_{HF}	0.86	/	0.64
R_{HF}	136	Ohm	73
Q_{MF}	8.48e-5	$F{\cdot}s^{a-1}$	2.41e-5
a_{MF}	0.66	/	0.16
R_{MF}	150	Ohm	23
Q_{LF}	2.24e-4	$F{\cdot}s^{a-1}$	1.4e-4
a_{LF}	0.46	/	0.07
R_{LF}	21	Ohm	13
Q_w	3.07e-2	$F{\cdot}s^{a-1}$	1.48e-2
a_w	0.29	/	0.15

Supplementary Table 7. Comparison of electrochemical performances of ASSBs based on halide electrolytes.

**Ref**[a]	**SE**	**First cycle capacity [mAh g^{-1}]**	**ICE[%]**[b]	**Cycling retention[%]**	**Current density [mA cm^{-2}]**
1	Li_2ZrCl_6	137(0.1C)	97.9(0.1C)	87.7(100 cycles 0.5C)	0.46(0.5C)
2	$Li_3YBr_3Cl_3$	124	91.9	88.7(60 cycles)	0.08(0.1C)
3	$Li_3InCl_{4.8}F_{1.2}$	160.6(4.47V)	92.0	63.5(70 cycles)	0.125(0.1C)
4	$SmCl_3·0.5Li_2ZrCl_6$	135(4.3V)	83.0	85(600 cycles)	0.405(0.5C)
5	ZrO_2-$2Li_2ZrCl_5F$	155(4.3V)	92.2	82.5(300 cycles)	0.58(0.5C)
6	$Li_{1.75}ZrCl_{4.75}O_{0.5}$	137.5(0.1C)	98.3(0.1C)	91.9(150 cycles, 5C)	3.85(5C)
This work	HE-LIC	144(0.1C)<br>132(0.5C)	97.0(0.1C)<br>93.3(0.5C)	88.9(500 cycles, 0.5C)	0.51(0.5C)
This work	HE-LIC	185(0.2 C, 4.6 V)	95.1	91.6(50 cycles)	0.29(0.2C)

[a] reference; [b] initial Coulombic Efficiency; Charge cut-off voltages of the unmarked cells are 4.2 V.

References

1 Wang, K. *et al.* A cost-effective and humidity-tolerant chloride solid electrolyte for lithium batteries. *Nat. Commun.* **12**, 4410 (2021).

2 Liu, Z. *et al.* High Ionic Conductivity Achieved in $Li_3Y(Br_3Cl_3)$ Mixed Halide Solid Electrolyte via Promoted Diffusion Pathways and Enhanced Grain Boundary. *ACS Energy Lett.* **6**, 298-304 (2021).

3 Zhang, S. *et al.* Advanced High‐Voltage All-Solid-State Li-Ion Batteries Enabled by a Dual-Halogen Solid Electrolyte. *Adv. Energy Mater.* **11** (2021).

4 Fu, J. *et al.* Superionic Conducting Halide Frameworks Enabled by Interface-Bonded Halides. *J. Am. Chem. Soc.* **145**, 2183–2194 (2022).

5 Kwak, H. *et al.* Boosting the interfacial superionic conduction of halide solid electrolytes for all-solid-state batteries. *Nat. Commun.* **14**, 2459 (2023).

6 Hu, L. *et al.* A cost-effective, ionically conductive and compressible oxychloride solid-state electrolyte for stable all-solid-state lithium-based batteries. *Nat. Commun.* **14** (2023).
